# Reports on Hospitals of the United Kingdom

**Published:** 1914-05-16

**Authors:** Henry Burdett


### REPORTS ON

### Hospitals of the United Kingdom.

By SIR HENRY BURDETT, K.C.B., K.C.V.O.

**SERIES III.**

### MARGATE COTTAGE HOSPITAL.

This Cottage Hospital was established in 1876, and was enlarged in 1898 as a Memorial to King Edward VII. As originally planned it was an attractive-looking and fairly typical institution of its kind. It consisted of a house or administration block, on either side of which was a ward for men and a ward for women. In 1898 it was enlarged, when the central block was practically rebuilt and the whole outward aspect of the building was changed.

### The Memorial Fund and the Medical Staff.

In 1912, the local committee, having £10,000 available for the King Edward Memorial, approached a firm of architects in London to prepare plans for the reconstruction of the hospital. These plans were not approved by the medical staff, who have wisely and properly expressed their opinion that the existing building should be demolished and an entirely new hospital erected on modern and up-to-date lines. The committee adopted the suggestions of the honorary medical staff, and sent for consideration to the local King Edward VII. Memorial Fund Committee plans and a scheme for an entirely new hospital, estimated to cost £13,000. This committee decided that the inadequate financial support given to the Memorial Fund made the scheme, however desirable, impossible of consummation so far as they were concerned, and the plans for a new hospital were therefore abandoned, at least temporarily.

### The Committee's Alternative Scheme.

These plans are the property of the Hospital Committee, which is not willing that they should be published with this scheme or report. It was estimated that a sum of £20,000 would be required to carry out these plans and thoroughly to furnish and equip the new hospital when erected. Consequently the King Edward Memorial Committee (local) intimated that, if the Hospital Committee would produce a satisfactory alternative smaller scheme, they would reconsider the matter and recommend their subscribers to allow their contributions to go towards the cost of carrying it out. Plans were then prepared for the extension of two wards and other additions, including further accommodation for the nurses, the estimated cost being about £2,500, inclusive of furniture. The number of beds in the hospital has thus been increased from twenty-two to thirty-one, including cots.

When we visited the hospital on November 3, 1913, we found the extended wards would each contain ten beds. It was intimated to us that there was a proposal to put twelve beds in each of these wards, but such a step would be unjustifiable having regard to the wall and floor space available. The hospital wards have no bath or lavatory accommodation in connection with them, these necessary adjuncts being quite separate from the wards.

### Unfortunate Additions.

Further, in the case of the women's ward they are so faulty that the bathroom not only contains the slop-sink but the water-closet as well. We, therefore, share the opinion of the committee that the alterations recently made must be regarded as of a temporary character, for they have not improved the buildings of the hospital, whilst they have left the old operation theatre and the other defective portions, such as the bath and lavatory accommodation, quite untouched. The recent additions have spoilt the small hospital which existed formerly, and the sooner the original recommendations of the medical staff are taken seriously in hand and an energetic and continuous effort is made to raise the balance of the £20,000 required to build and equip a modern and up-to-date hospital, the better for the reputation of the citizens and for the sick and injured persons in Margate and the district who may require hospital care.

### The Lesson of an Accident Case.

Whilst we were visiting the hospital, an accident case was brought in which illustrated how defective the present entrance up three steps to the hospital in fact is. We would suggest that the steps should be removed, and that the hospital should be approached by a gentle slope rising from the pavement to the floor level of the entrance hall. This can readily be done at small cost, and it is evident that the need for this improvement is urgent in the interests of those who may be brought to the hospital suffering from accidents.

The matron was not in the hospital the day we called, she having been away ill for several months, and the sister in charge was out. We were much indebted to the courtesy of a trained nurse, temporarily engaged, who took us over the buildings.

### ROYAL SEA BATHING HOSPITAL, MARGATE.

We inspected this hospital on November 3, 1913, and found it very defective in ward, kitchen, and other accommodation. The nurses seemed to us to be far too few in number, having regard to the fact that as many as 187 patients are crowded into the wards and verandahs of this institution during several months of the year. The hospital has a mortuary with a chapel, but it requires redecoration and was not too well kept, probably because there are so few deaths at this hospital. The assistant matron took us round, in the absence of the matron, and we asked her to tell the matron that we should be glad to have full particulars of the requirements or needs of the hospital, as she found them to exist.

The patients had a remarkably healthy appearance and were cheerful and bright. It appeared probable that trouble is caused by the admission of a number of young men, who are apt to get a little out of hand, due, no doubt, to the invigorating character of the air. The present condition of this hospital requires to be gone thoroughly into, and the time is approaching, if it has not already arrived, when the whole equipment of the place should be thoroughly overhauled and improved.

### UCKFIELD COTTAGE HOSPITAL.

This hospital, which was established in 1881 and enlarged in 1913, consists of a villa residence containing eight beds where there is still room for more. Two large rooms were added in 1913, chiefly with the object of giving the matron a new bedroom. The hospital contained only one patient when we visited it on October 31, 1913. The register indicated that there were thirty-nine patients in 1913, that the illnesses were slight, and that the three most serious cases were sent to the Sussex County Hospital, Brighton, for which this institution appears to be a sort of receiving station. Unless the medical staff is strengthened, we presume, in view of the changes which are pending in respect of institutional treatment, this is an institution which is likely to disappear.

